# Pharmacological therapies for infantile hemangiomas: A clinical study in 853 consecutive patients using a standard treatment algorithm

**DOI:** 10.1038/srep21670

**Published:** 2016-02-15

**Authors:** Ling Zhang, Wei-En Yuan, Jia-Wei Zheng

**Affiliations:** 1 Department of Oral-maxillary Head and Neck Surgery, College of Stomatology, Ninth People’s Hospital, Shanghai Jiao Tong University School of Medicine; Shanghai Key Laboratory of Stomatology. Shanghai 200011, China; 2 School of Pharmacy, Shanghai Jiao Tong University, Shanghai, People’s Republic of China

## Abstract

Infantile hemangiomas are the most common infantile benign vascular tumor. While most infantile hemangiomas proliferate then involute, some may persist and require treatment for reasons including risk of disfigurement or functional impairment. Treatments currently include observation, pharmacological therapy, laser, cryosurgery, surgery and radiotherapy. Although pharmacological therapy is a well accepted treatment option, limited studies have evaluated the efficacy of different drug therapies. In this study, we compare different pharmacological modalities in the management of infantile hemangiomas. The study included 853 infants with proliferative infantile hemangiomas who were treated with topical timolol, oral propranolol, intralesional pingyangmycin, or intravenous vincristine from 2009 to 2012. Treatment stratification was based on clinical severity of the tumor. Response to the treatment was clinically evaluated and graded as: excellent, good, poor, or no response. Response to pharmacological therapies was excellent in almost all infantile hemangiomas. In addition, patients younger than 8 months responded highly to pharmacological treatment (89.1%), while patients older than 8 months were less responsive to treatment (36.3%). There were no instances of life-threatening complications. Overall, these findings support the efficacy of timolol, propranolol, pingyangmycin and vincristine in the treatment of infantile hemangiomas, especially in the youngest patient cohort (8 months or younger).

Infantile hemangioma has a characteristic clinical appearance with approximately 60% of cases arising in the head and neck^1^. These tumors undergo a periods of rapid proliferation in the first year of life; then followed by a slow involution phase^2^. In the past, the treatment of infantile hemangiomas commonly involved implementing a “watch-and-wait” strategy. However, such an approach may not be suitable for all cases. In addition, adverse outcomes such as ulceration, scar formation, disfigurement and possibly life threatening may occur^3^. Therefore, intervention instead of observational therapy may be more appropriate for aggressive lesions.

Previous work has demonstrated that the β-blocker propranolol has an inhibitory effect on infantile hemangiomas^4^. Propranolol is a highly effective and relatively safe drug, and has recently replaced corticosteroids as the first-line treatment for infantile hemangiomas. Topical timolol, another β-blocker agent, may be considered to avoid any potential adverse effects of systemic propranolol^5^. However, β-blockers are not universally effective in controlling all infantile hemangiomas. For patients who have not responded well to these primary treatments, intralesional injections of pingyangmycin have proven to be clinically efficacious^6^. Similar to bleomycin A5, pingyangmycin is commonly used as chemotherapeutic drug but more recently has been used as a sclerosant for the treatment of vascular anomalies. In patients with complicated hemangiomas who have failed other primary treatments, the chemotherapeutic drug, vincristine, is a viable treatment option. When used at low doses, vincristine is reported to be effective in treating a majority of patients with severe, potentially function-altering or life threatening infantile hemangiomas^7^. To date, while there are many studies that discuss the efficacy of single drugs, there are few studies that evaluates multiple therapeutic agents. In this study, we reveal the results of treating patients with infantile hemangiomas with timolol, propranolol, pingyangmycin and vincristine, and in turn, propose a comprehensive treatment algorithm for infantile hemangiomas.

## Methods

### Data collection

This clinical research was approved by the Ninth People’s Hospital, Shanghai Jiao Tong University School of Medicine China. The human subject protocol was approved by the Committee on Clinical Investigation. Clinical diagnoses of infantile hemangioma were confirmed by the Department of Oral-maxillary, head and neck surgery at Ninth People’s Hospital Shanghai. Informed consent was obtained in accordance with The World Medical Association Declaration of Helsinki. All the following methods were also carried out in accordance with the approved guidelines (http://jama.jamanetwork.com/article.aspx?articleid=1760318).

853 patients with proliferating infantile hemangiomas were referred for pharmacological therapy over a time period spanning from January 2009 to December 2012. A standardized database recorded patient details, which included patient gender, age at treatment initiation, location and size of the lesion, treatment dose, clinical response, complications resulting from treatment and follow-up.

### Treatment Algorithm

Patients were treated (as needed and per detailed criteria) with agents as sequentially listed below. For β-blocker and pingyangcymin therapy, patients were treated on an outpatient basis. The methods were carried out in clinical diagnoses of infantile hemangioma with approved the guidelines which 0.5% (w/w) timolol maleate was applied topically to the His; propranolol dosing was orally; pingyangmycin was intralesional injection and vincristine was intravenous^8^.

### Timolol maleate dosing

As an initial treatment for patients with cutaneous infantile hemangiomas with a diameter less than 1.0 cm, a 0.5% solution of timolol maleate was applied topically over the lesion surface using a cotton swab three times daily. The mean time of treatment was 8 months.

### Propranolol dosing

If no response was observed with timolol following a two week treatment period, oral propranolol was administered twice daily at a daily total dose of 2 mg/kg. The mean time of treatment was 6 months. Prior to propranolol treatment, an electrocardiogram was performed to detect any possible congenital cardiac abnormalities. There were three key points that were relayed to the parents of the patients: (1) Propranolol must be administered following a meal due to risk of hypoglycemia; (2) In the instance of any bronchial hyperreactivity/bronchospasm, treatment must be terminated; and (3) Treatment may be resumed after symptoms have subsided.

The dosage was adjusted for any increases in weight observed during treatment. Serial photographs of the infantile hemangiomas and ultrasound were taken in order to assess the efficacy of propranolol treatment at two weeks, one month and three months following treatment.

### Pingyangmycin dosing

For patients who did not respond well to propranolol or whose lesions were already in the involution period with a diameter equal or less than 3 cm, intralesional injections of pingyangmycin (8 mg/ampoule, Tianjin Tai-He Pharmaceutical, Tianjin,China; dissolved in 8ml of saline solution, for a final concentration of 1 mg/ml) were administered. To evaluate for any potential allergic reactions to treatment, patients were observed for fifteen minutes following injection for any signs of reaction. To minimize risk of pulmonary fibrosis, treatments were performed at three week intervals and continued until the surface of the lesions paled in appearance. The maximum dose was no greater than 2 mg per injection, and the total dose never exceeded 16 mg for any patient. The mean time of treatment was 2.5 months. For the potential late adverse effect, 1 year follow up was performed.

### Vincristine dosing

For patients with complicated hemangiomas (diameter **> **5 cm) who failed propranolol, intravenous vincristine was administered at a dose of 1.0 mg/m^2^ every two weeks. Each round of treatment consisted of 12 weeks, with an interval of 1 week between rounds and a total of 2 rounds. Those patients treated intravenously with vincristine were hospitalized for one to two days for observation. Patients were monitored closely for the development of any potential complications, and dosage was adjusted according to the progression of the hemangioma. The mean time of treatment was 4.5 months.

### Therapy response evaluation

Photos were taken prior to treatment as well as at each follow-up visit. A pediatric oncologist evaluated the therapeutic response. Therapeutic response was graded as follows: a tumor volume reduction greater than 75% based on ultrasound was considered an ‘excellent’ response, a reduction greater than 50%, a ‘good’ response, a reduction of 25% or less, a ‘poor’ response, and a 0% reduction, ‘non-responsive.’ Ultrasound was also used to evaluate the depth of the lesion.

## Results

### Demographic data

A total of 853 patients were examined in this study. The patients’ mean age was 6.98 ± 6.74 months and the overall male-to-female ratio was 1:5.1. 15.4% (131) of infants were premature and 30.4% (259) of mothers had a history of treatment with a tocolytic agent during the gestational period. Of these agents, progesterone was the most common. In 55% (469) of the patients, hemangiomas were localized on the left side of the face and neck, whereas in the remaining 45% (384) of patients, the lesion was localized on the right side of the face and neck. The most common anatomic site was lips (approximately 11.2%; 96) and the eyelids (approximately 10.2%; 87) (Table 1). The mean hemangioma size was 21.14 ± 20.83 cm^2^. Approximately 95.8% (817) of the hemangiomas were superficial in type; 3.1% (27) were subcutaneous and 1.1% (9) were mixed.

### Clinical response rate

Any clinical response to any drug therapy in this study was high for the superficial infantile hemangiomas 98.5% (805; p < 0.05) and 81.5% (22; p < 0.05) for subcutaneous infantile hemangiomas. The clinical response rate in 787 patients younger than 8 months was 92.3%, The clinical response rate was 59.1% in 66 patients older than 8 months. This difference was statistically significant (P < 0.001) and was not related to gender. Drug treatment is the most efficacious when tumors were found in the parotid region; whereas, the least efficacious in those were found in the lip region. The response rate was 96.6% (85/88) and 80.1% (109/136), respectively.

### Timolol response rate

**Figure 1:**
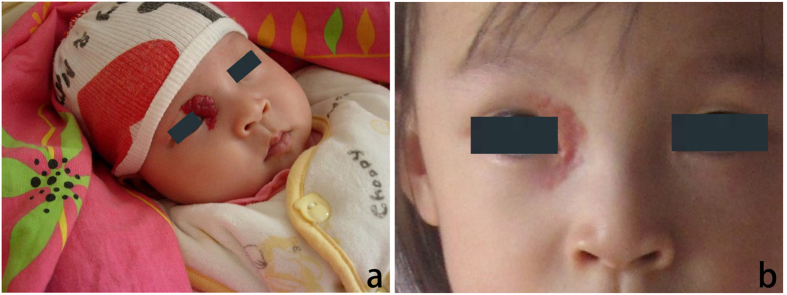
(**a**) A 6-month-old female patient with a hemangioma located on her right endocanthion. (**b**) Patient seen at 14 months following treatment with 0.5% timolol maleate solution applied topically over the surface of the hemangioma. Note the dramatic reduction in the size of the hemangioma.

10.2% (87) of patients were treated with timolol. In this group, response rates were as follows: 25.3% (22) ‘excellent’; 39.1% (34) ‘good’; 21.8% (19) ‘ poor’; and 13.8% (12) ‘non-responsive’ (Fig. 1a,b).

### Propanolol response rate

**Figure 2:**
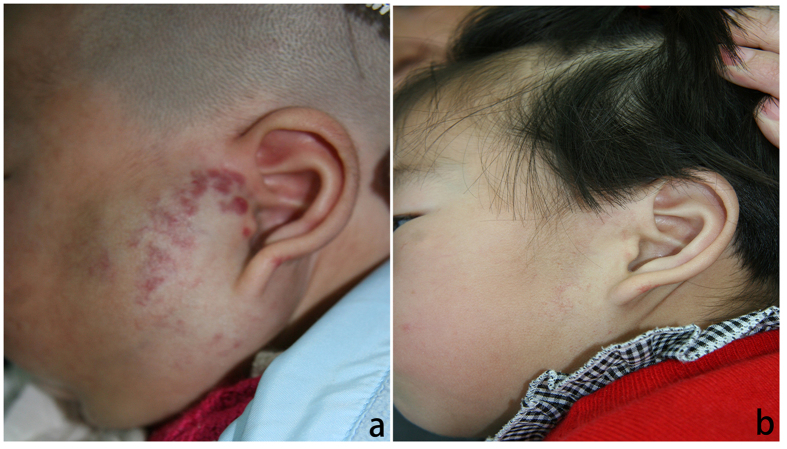
(**a**) A 3-month-old male patient with a large hemangioma located over the left parotid gland. (**b**) The same patient at 10 months following treatment with oral propranolol. Note that the hemangioma is almost completely resolved.

93.4% (797) of patients were treated with propranolol (including patients that exhibited either a poor response, or no response to timolol treatment). In this group, response rates were as follows: 29.5% (235) ‘excellent’; 57.3% (457) ‘good’; and 10.8% (86) ‘poor’; and 2.4% (19) ‘non-responsive’ (Fig. 2a,b).

### Pingyangmycin response rate

**Figure 3:**
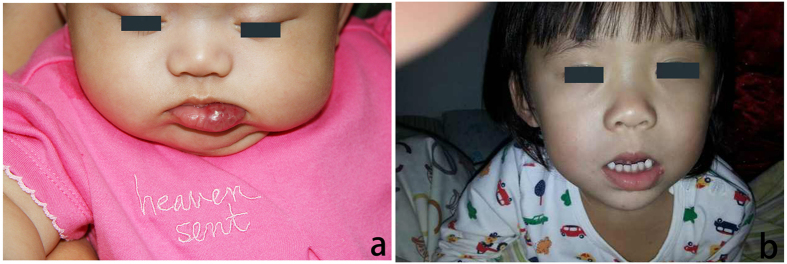
(**a**) An 8-month-old female patient with a hemangioma located on the lip. (**b**) The same patient seen at 22 months following intralesional pingyangmycin injections. Note that the major extent of the lesion has disappeared.

Within the non-responsive propranolol patient cohort (19), 11 began pingyangmycin injections treatment (Fig. 3a,b). As per inclusion criteria, the diameter of the lesions were no more than 3 cm. Response rates were as follows: 45.5% (5) ‘excellent’; 36.3% (4) ‘good’; and 18.2% (2) ‘non-responsive’. Of the non-responsive group, one patient was fourteen months old with the lesion located in the right neck, and the other patient was fifteen months old with the lesion located in the lower lip.

### Vincristine response rate

**Figure 4:**
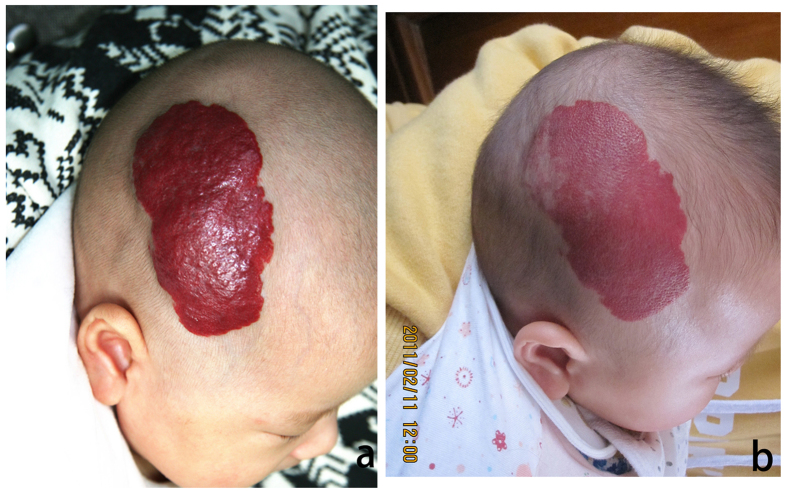
(**a**) A 6-month-old male patient with hemangioma located on the head. (**b**) Same patient seen after 2 months while undergoing treatment with vincristine. Note that the lesion is already markedly diminished at this stage of treatment and treatment is ongoing.

The remaining 8 non-responsive patients treated with propranolol exhibited complex tumors (mean diameter > 3.0 cm or multiple lesions) and were treated with vincristine after oral propranolol administration was deemed unsuccessful (Fig. 4a,b). Response rates were as follows: 37.5% (3) ‘excellent’; 62.5% (5) ‘good’.

**Figure 5:**
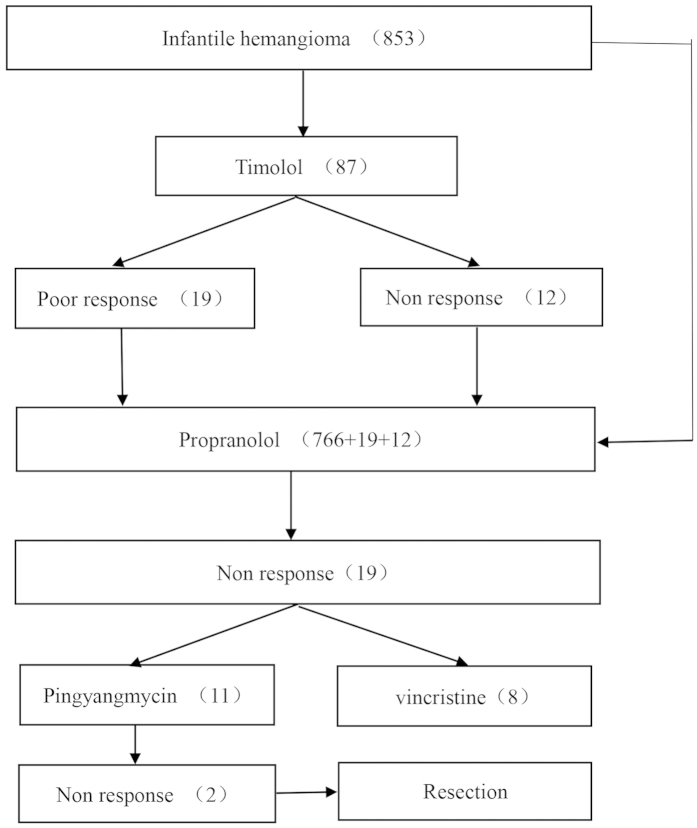
Chart of treatment assignments for patients treated at our center.

**Figure 6:**
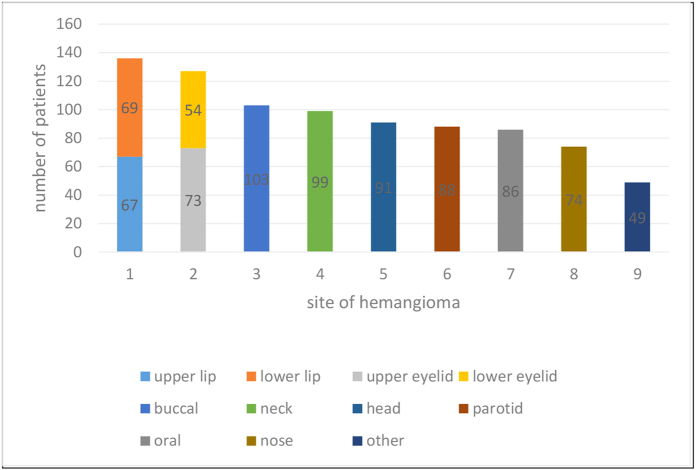
Hemangioma anatomic site.

Overall, 17 of the 19 propranolol ‘non-responsive’ patients showed satisfactory results to additional advanced treatment. Two patients from the pingyangmycin-treated group required surgical intervention. There were no life-threatening complications in this study. Cases of minor complications were low; 0.59% (5) and 0.35% (3) of patients treated with propranolol displayed symptoms of diarrhea and bronchospasms, respectively. The mean duration for follow-up was 2.43 ± 1.08 years (Figs 5 and 6).

## Discussion

Infantile hemangiomas are benign vascular neoplasms with a characteristic clinical appearance. They generally appear as red spots after birth then enter a stage of rapid proliferation for several months in the first year, typically followed by spontaneous involution^9^. However, up to 20% of lesions require immediate intervention due to complications such as bleeding, deformity, ulceration and rarely, fatality^10^. The therapeutic effect of the non-selective β-adrenergic antagonist propranolol in treating infantile hemangiomas was first reported in 2008^11^. At present, propranolol is commonly used as a first line treatment for infantile hemangiomas^12^. An alternative approach to treating infantile hemangiomas is the topical application of another β-blocker, timolol maleate, (0.5%)^13^, particularly in cases of periocular hemangioma^14^. This therapy has the added benefit of avoiding any potentially adverse systemic effects which can arise from oral propranolol administration. In this study, we applied a timolol maleate solution over the surface of lesions with a diameter less than 1.0 cm. Our findings suggest that topical timolol is a safe and effective treatment for the majority of superficial hemangiomas. However, thicker or deeper lesions require a greater depth of penetration than topical application can allow.

In cases where timolol treatment was ineffective or the tumor diameter was greater than 1.0 cm, patients were treated orally with propranolol. In a prior study, we treated 40 infants with oral propranolol, administered at a dose of 2 mg/kg body weight per day in 2 divided doses. These infants were admitted as inpatients, and blood pressure, heart rate and blood glucose levels were monitored closely at 24 hrs, 48 hrs, 72 hrs, 96 hrs and 108 hrs after initiating propranolol treatment. There was no observable impairment drop in blood pressure or heart rate observed. Therefore, in this study, the patients subjected to oral propranolol were treated as outpatients following a simple examination. Only 8 patients exhibited some minor side effects (diarrhea and bronchospasms), however, stopping the treatment, these patients had not received any additional treatment, the symptoms were alleviated, and when treatment was resumed, the side effects were no longer observed for the patients treated in this study. It is important to note that when pediatricians prescribe propranolol at a dosage that exceeds 4 mg/kg per day, it can put the infant patient at risk for developing hypoglycemia^15^,^16^. Therefore, we believe that the propranolol dosage of 2 mg/kg per day used in this study is well within a safe range. The duration of propranolol therapy lasted an average of 6 months, and treatment was terminated after the infants reached 1year of age. In the majority of cases, the lesions faded. However, propranolol was not successful in treating all infantile hemangiomas. For those patients who did not respond well to propranolol, we alternatively began treating with pingyangmycin, a sclerosing agent that destroys endothelial cells. We observed that sclerotherapy was effective for small and middle-sized infantile hemangiomas (diameter < 3.0 cm). It should be noted that when the cumulative dose of pingyangmycin is greater than 160 mg, there is a risk for potential pulmonary fibrosis^17^. However, in our study, the total dose of pingyangmycin administered was always less than 16 mg. Furthermore, there were no systemic toxicities observed in any of the patients treated with pingyangmycin. For involuting hemangiomas, local injection of pingyangmycin succeeded in shortening the involution process. We did have some patients present with large hemangiomas, which can cause functional impairment, and even pose a threat to life. In these cases, it was most beneficial for the patients to receive vincristine treatment for the lesions^18^. Vincristine was administered intravenously at a dose of 1.0 mg/m^2^. Following two rounds of treatment, the majority of patients responded well to vincristine therapy. When used as a chemotherapeutic agent, vincristine has many side effects, including constipation, neuropathy, immunosuppression, and alopecia^19^. It is encouraging that at our administered vincristine therapeutic dose, side effects including neurotoxicity appears to be extremely limited.

## Conclusions

**Figure 7:**
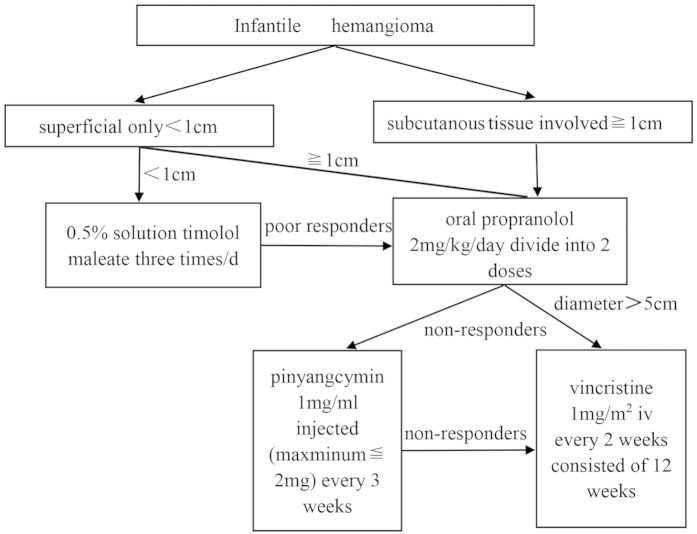
Treatment algorithm for infantile hemangiomas.

At present, there is no single drug that can be effective and universal treatment for all hemangiomas^20^. This is largely due to the diverse presentation of infantile hemangiomas in patients. In our study, which evaluates an extensive and diverse range of infantile hemangioma cases, we have demonstrated that the successful treatment of infantile hemangioma is contingent upon individual assessment and selection of an appropriate therapeutic strategy. Moreover, we also show here that early intervention is critical in treating infantile hemangiomas. This is especially relevant in cases of complex infantile hemangiomas or those which may arise in locations that can impair functionality, for example, the periocular region or within the airways. Future research and development in this field should focus on the development of treatment strategies which are timely and aimed at the individual. This would ideally involve the coordinated input from a multidisciplinary vascular anomaly team, including specialists such as otolaryngologists, ophthalmologists and dermatologists, as required (Fig. 7).

## Additional Information

**How to cite this article**: Zhang, L. *et al.* Pharmacological therapies for infantile hemangiomas: A clinical study in 853 consecutive patients using a standard treatment algorithm. *Sci. Rep.* **6**, 21670; doi: 10.1038/srep21670 (2016).

## Figures and Tables

**Table 1:** Patient characteristics by treatment group, 1 = timolol, 2 = propranolol, 3 = pingyangmycin, 4 = vincristine.

	Group1	Group2	Group3	Group4
Quantity	87	747	11	8
Gender (M/F)	16/71	147/600	2/9	2/6
Mean age (m)	1.91 ± 1.75	5.23 ± 1.94	12.43 ± 2.56	6.21 ± 3.32
Diameter (cm)	0–1.0	>1.0	1.0–3.0	>3.0
Medicine dose	3 drop	2.0 mg/kg	0–2.0 mg	1.0 mg/m2
Response rate	86.2%	97.5%	100%	89.5%
Time of treatment (m)	8	6	2.5	4.5
Complications	0	0.94% (8)	0	0
Follow-up (m)	24	20	22	18
